# Supplementary material for: Fe3O4 Hollow Nanosphere-Coated Spherical-Graphite Composites: A High-Rate Capacity and Ultra-Long Cycle Life Anode Material for Lithium Ion Batteries
Source: Nanomaterials (Basel). 2019 Jul 10;9(7):996. doi: 10.3390/nano9070996 (PMC6669536; doi:10.3390/nano9070996)
Supplement: Supplementary file 1 [file nanomaterials-09-00996-s001.pdf]

nanomaterials

Supplementary Materials

Fe_3O_4 Hollow Nanosphere-Coated Spherical-Graphite Composites: A High-Rate Capacity and Ultra-Long Cycle Life Anode Material for Lithium Ion Batteries

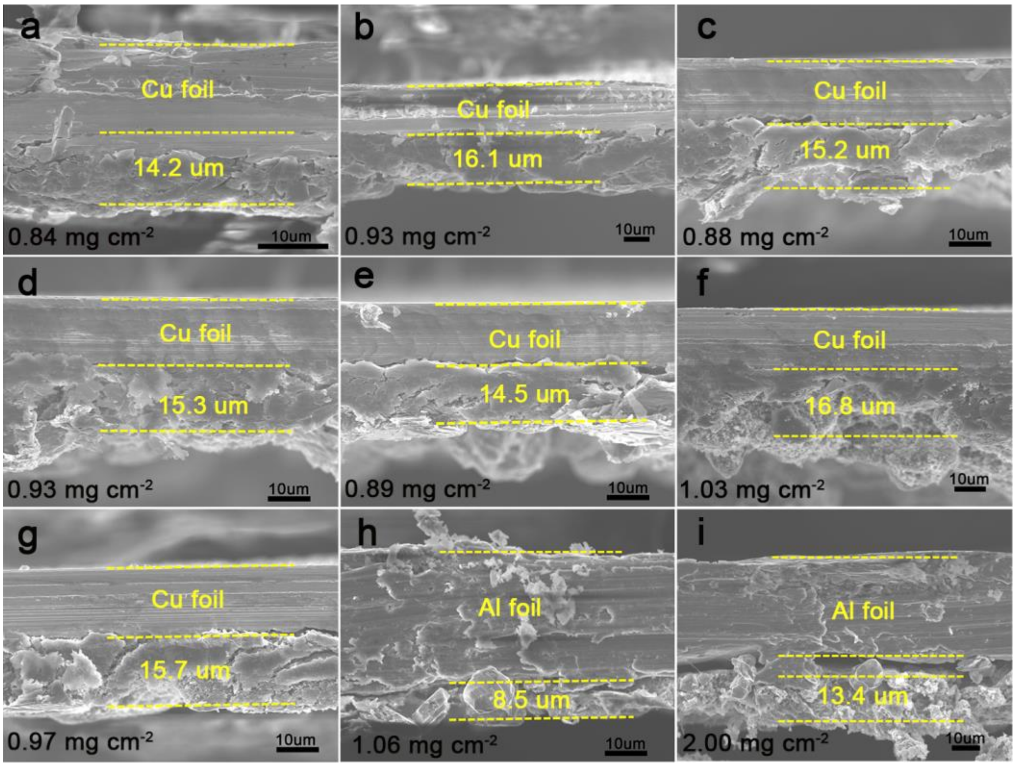

Figure S1. The loading weights and thickness of (**a**) SG, (**b**) SGO, (**c**) SGO/Fe_3O_4-1, (**d**) SGO/Fe_3O_4-2, (**e**) SGO/Fe_3O_4-3, (**f**) SGO/Fe_3O_4-4, (**g**) SGO/Fe_3O_4-5, (**h**) $LiCoO_2$-1 (matching with SGO) and (**i**) $LiCoO_2$-2 (matching with SGO/Fe_3O_4-4) electrodes, respectively.

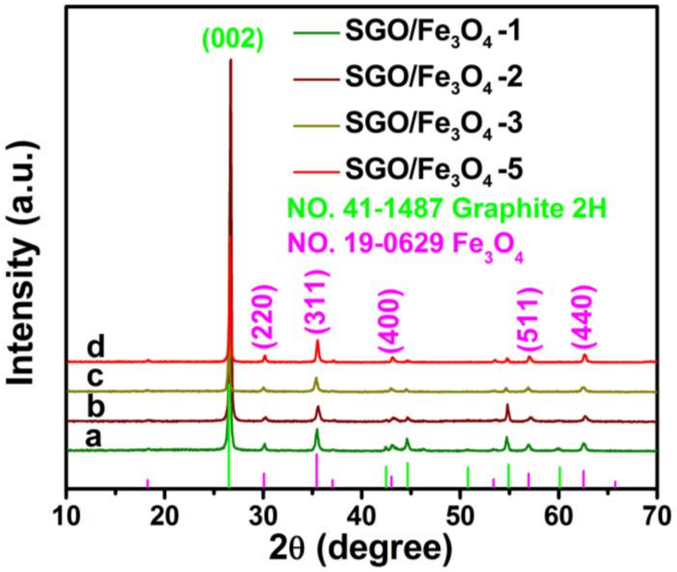

Figure S2. XRD patterns of different composites.

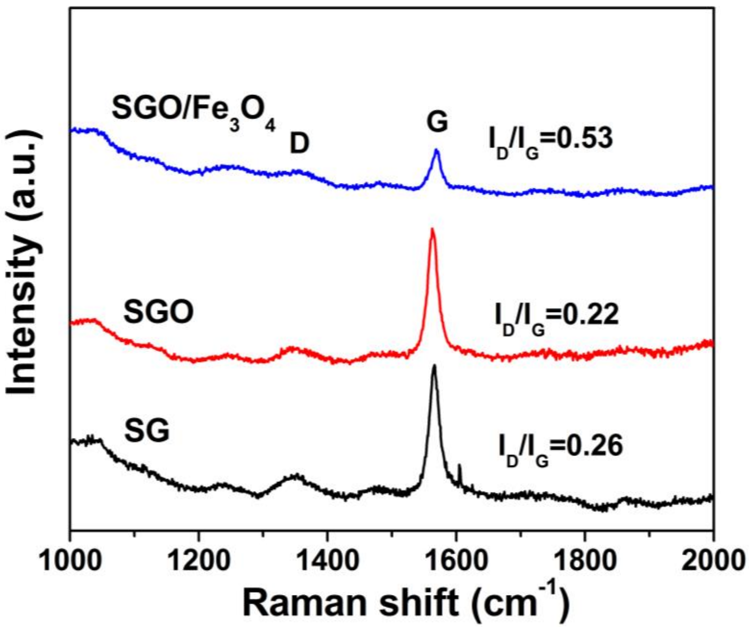

Figure S3. Raman spectra of three samples.

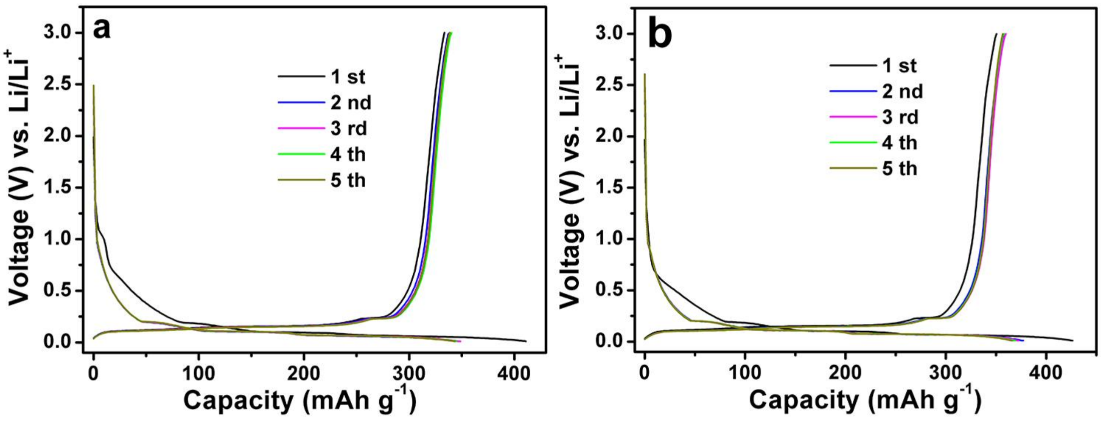

Figure S4. Galvanostatic lithiation/delithiation curves of (**a**) SG and (**b**) SGO at 0.1 A g^{-1}.

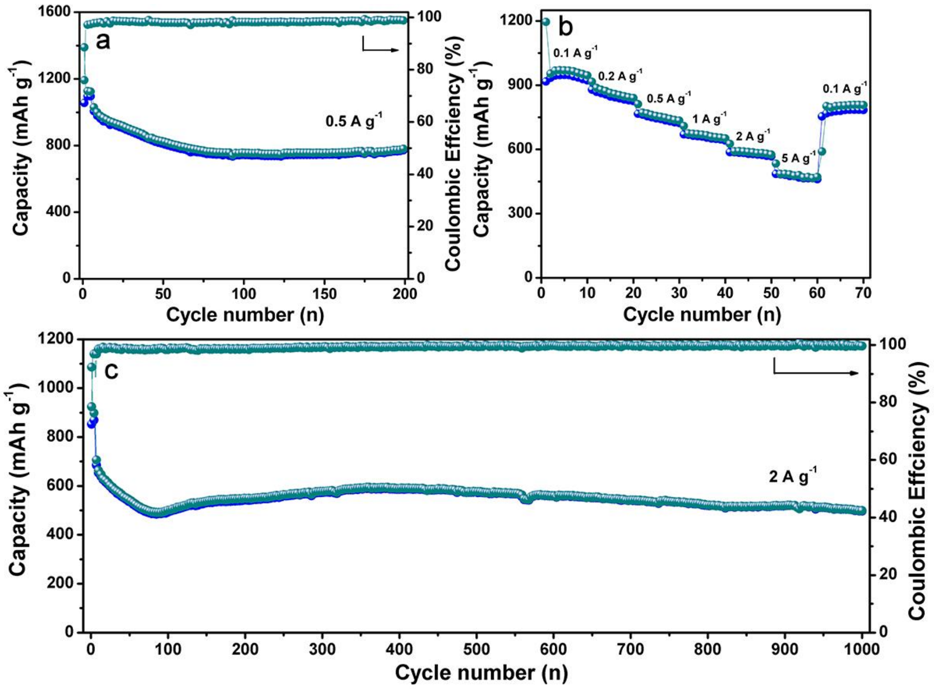

Figure S5. (**a**) Cycling performance at 0.5 A g^{-1}, (**b**) rate performance, and (**c**) long term cycling performance at 2 A g^{-1} of Fe_3O_4 hollow nanospheres.

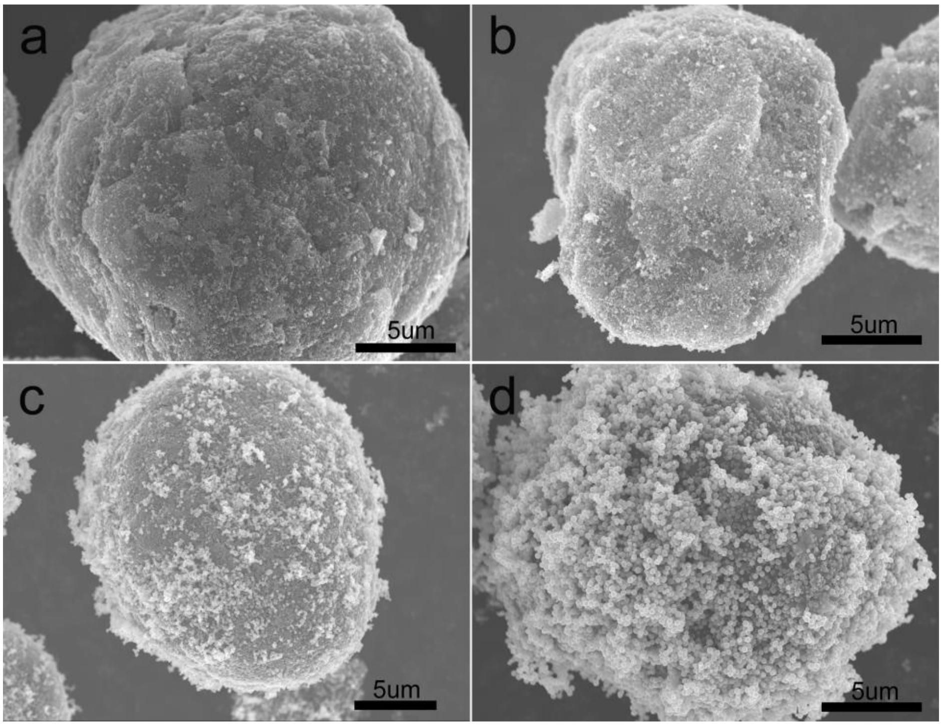

Figure S6. SEM images of (**a**) SGO/Fe_3O_4-1, (**b**) SGO/Fe_3O_4-2, (**c**) SGO/ Fe_3O_4-3, (**d**) SGO/Fe_3O_4-5 composites.

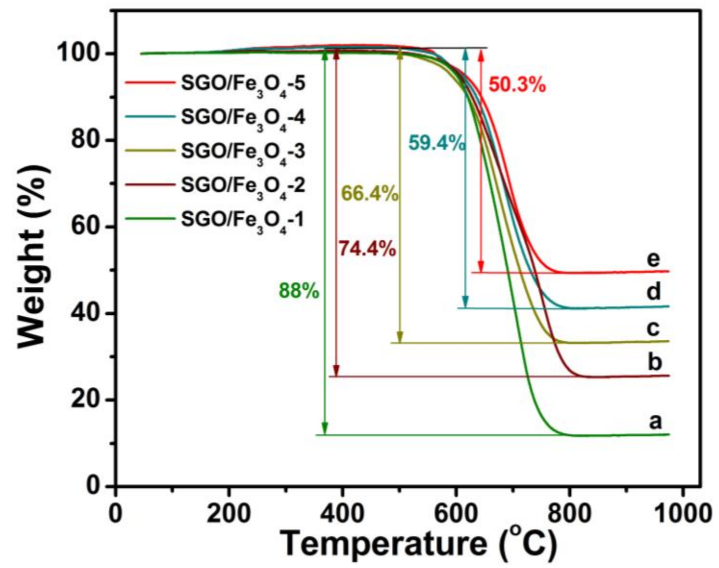

Figure S7. TG curves of different composites.

Table s1. Electrochemical impedance fitted parameters for Figure 6.

	Before cycling		After cycling		
	Rs	R(sf+ct)	Rs	Rsf	Rct
SG	2.29	404.46	2.66	13.07	41.75
SGO	0.97	158.99	4.72	34.69	33.36
SGO/Fe_3O_4-4	1.44	98.12	2.2	19.42	19.6
